# Why Some Plant Species Are Rare

**DOI:** 10.1371/journal.pone.0102674

**Published:** 2014-07-23

**Authors:** G. W. Weiger Wamelink, Paul W. Goedhart, Josep Y. Frissel

**Affiliations:** 1 Alterra, Wageningen UR, Wageningen, the Netherlands; 2 Biometris PRI, Wageningen UR, Wageningen, the Netherlands; University Copenhagen, Denmark

## Abstract

Biodiversity, including plant species diversity, is threatened worldwide as a result of anthropogenic pressures such as an increase of pollutants and climate change. Rare species in particular are on the verge of becoming extinct. It is still unclear as to why some plant species are rare and others are not. Are they rare due to: intrinsic reasons, dispersal capacity, the effects of management or abiotic circumstances? Habitat preference of rare plant species may play an important role in determining why some species are rare. Based on an extensive data set of soil parameters we investigated if rarity is due to a narrow habitat preference for abiotic soil parameters. For 23 different abiotic soil parameters, of which the most influential were groundwater-table, soil-pH and nutrient-contents, we estimated species responses for common and rare species. Based on the responses per species we calculated the range of occurrence, the range between the 5 and 95 percentile of the response curve giving the habitat preference. Subsequently, we calculated the average response range for common and rare species. In addition, we designed a new graphic in order to provide a better means for presentation of the results. The habitat preferences of rare species for abiotic soil conditions are significantly narrower than for common species. Twenty of the twenty-three abiotic parameters showed on average significantly narrower habitat preferences for rare species than for common species; none of the abiotic parameters showed on average a narrower habitat preference for common species. The results have major implications for the conservation of rare plant species; accordingly management and nature development should be focussed on the maintenance and creation of a broad range of environmental conditions, so that the requirements of rare species are met. The conservation of (abiotic) gradients within ecosystems is particularly important for preserving rare species.

## Introduction

Biodiversity, including plant species richness, is threatened worldwide [1], [2], [3], as a result of anthropogenic pressures such as an increase of pollutants [4] and climate change [5]. Rare species in particular are on the verge of becoming extinct. It is still unclear as to why some plant species are rare and others are not [6], [7], [8], [9]. Are they rare due to intrinsic reasons, dispersal capacity, the effects of management or abiotic circumstances? Habitat preference of rare plant species may play an important role in determining why some species are rare and others are not.

Species occurrence can also be limited by dispersal capacity, for instance when all habitat requirements are met but the species is not yet able to reach suitable habitat [10], [11], [12]. Human influence is another major factor that impacts on the occurrence of rare species; for example, through changes in habitats caused by construction of infrastructure and built development, water-related management or intensified agricultural land use [10], [13], [14], [15], [16]. Nature management can be applied to conserve the habitats necessary for the rare species and thus prevent them from becoming extinct [16], [17]. Understanding the differences in habitat requirements between rare species and common species is also likely to be an important factor in protecting rare species [6].

All plant species establish their own niches in their preferential habitat and this is a major determinant of their spatial distribution. The occurrence of species is being determined at different scales, ranging from the biogeographical till the habitat scale. On the biogeographical scale species occurrence is probably mostly limited by climatic parameters such as temperature and precipitation. The realized distribution of the biogeographical niche is often referred to as the climate envelope of a species and used to predict the effects of climate change on species occurrence [12], [18]. Within the climate envelope some habitats are suitable and some are not. Species occurrence on the habitat scale can be limited by factors such as the vegetation types that are present as a result of management, the absence of suitable abiotic conditions including soil pH and nutrient availability, the status of the groundwater table (both water quality and availability) or fine scale heterogeneity in for example vegetation structure, soil gradients or management related gradients [17], [19], [20].

The specific topic of this paper is to consider the influence of abiotic (soil) conditions in relation to rarity of species. We hypothesised that rare species have narrower habitat preferences for abiotic soil conditions than common species. Based on an extensive data set of measured soil parameters we therefore investigated if rarity of plant species is constrained by their restricted habitat preference for abiotic soil parameter.

## Material and Methods

### Database and abiotic soil parameter selection

Species responses for abiotic soil parameters were estimated based on field measurements in just over 8000 plots in the Netherlands, mostly collected from literature. For each plot species composition was recorded and a mixed soil sample was taken from the upper soil layer (mostly upper 10 cm) and analysed in a laboratory. Plot sizes ranged from 1 m^2^ for grasslands till 100 m^2^ for forest. Plots were recorded following the Brown-Blanquet method [21]. The abiotic values of the plot were linked to all the species in the plot, including mosses, lichens shrubs and trees. The data cover the period from 1936 till present day and the database is still expanding. Each plot is accompanied by at least one abiotic soil measurement (e.g. pH or nutrient availability). The most frequently measured soil parameter is pH, with well over 5,000 entries. The database is part of the European metadata database for vegetation plots (the Ecological Conditions Database; GIVD ID EU-00-006) [22].

Abiotic ranges for an individual species were only estimated when at least 25 positive findings in relation to the abiotic parameter combination were present in the database. The procedure was carried out for the 23 different soil parameters (Table 1).

**Table 1:** Species ranges for 23 abiotic soil parameters for rare and common species, including standard error, the difference between both groups and the p-values for the difference (two tail student t-test, n.s.; not significant, * p<0.05, ** p<0.01, *** p<0.001).

Parameter	unit	Common species		rare species		Δ	against rare	p
		range	s.e.	range	s.e.		%	
C/N	-	4.95	1.97	4.47	2.19	0.48	10.63	0.004^**^
C_total_	mg/kg	10.92	3.80	9.80	3.59	1.12	11.45	0.000^***^
Ca_H2O_ 1	mg/kg	5092.36	2028.77	4707.72	1865.53	384.64	8.17	0.017^*^
CaCO_3_	mg/kg	2.77	1.11	2.48	1.29	0.29	11.86	0.002^**^
Cl	mg/kg	209.86	199.28	168.42	199.15	41.44	24.60	0.010^*^
Electronic conductivity	mS	518.06	205.58	481.61	219.12	36.45	7.57	0.031^*^
K_H2O_ 1	mg/kg	102.05	32.03	87.47	27.61	14.58	16.67	0.000^***^
Mg_H2O_ 1	mg/kg	151.42	62.91	142.84	66.85	8.58	6.01	0.096^n.s.^
MHG^2^	cm -bs	47.74	16.19	47.50	19.57	0.24	0.51	0.860^n.s.^
MLG^3^	cm -bs	57.54	21.59	56.01	27.20	1.52	2.72	0.409^n.s.^
MSG^4^	cm -bs	40.38	12.16	34.44	10.86	5.94	17.24	0.000^***^
Moisture	%	19.36	7.08	18.00	7.14	1.36	7.57	0.018^*^
Na_CaCl2_ 5	mg/kg	56.88	30.22	47.67	28.05	9.20	19.31	0.000^***^
Na_H2O_ 1	mg/kg	263.51	142.98	209.74	131.69	53.78	25.64	0.000^***^
NH4_CaCl2_ 5	mg/kg	6.64	2.61	5.67	2.61	0.96	16.96	0.000^***^
NO3_CaCl2_ 5	mg/kg	19.72	6.96	13.69	7.27	6.03	44.03	0.000^***^
N_total_	mg/kg	3896.27	1845.54	3467.11	1526.65	429.16	12.38	0.003^**^
Organic matter	mg/kg	9.04	4.33	8.43	4.41	0.60	7.16	0.086^n.s.^
P_CaCl2_ 5	mg/kg	1.62	0.66	1.25	0.73	0.37	29.20	0.000^***^
P_citric acid_	mg/kg	290.97	99.87	218.46	82.64	72.52	33.19	0.000^***^
P_total_	mg/kg	434.98	167.16	359.89	162.22	75.09	20.86	0.000^***^
pH_H2O_	-	1.16	0.41	0.96	0.42	0.20	20.88	0.000^***^
pH_KCl_	-	1.18	0.49	0.99	0.49	0.18	18.65	0.000^***^

Results per species are given in Appendix S1.

1 In water extract,

2 mean highest groundwater level,

3 mean lowest groundwater level,

4 mean spring groundwater level,

5 in CaCl_2_ extract.

### Species response

**Figure 1:**
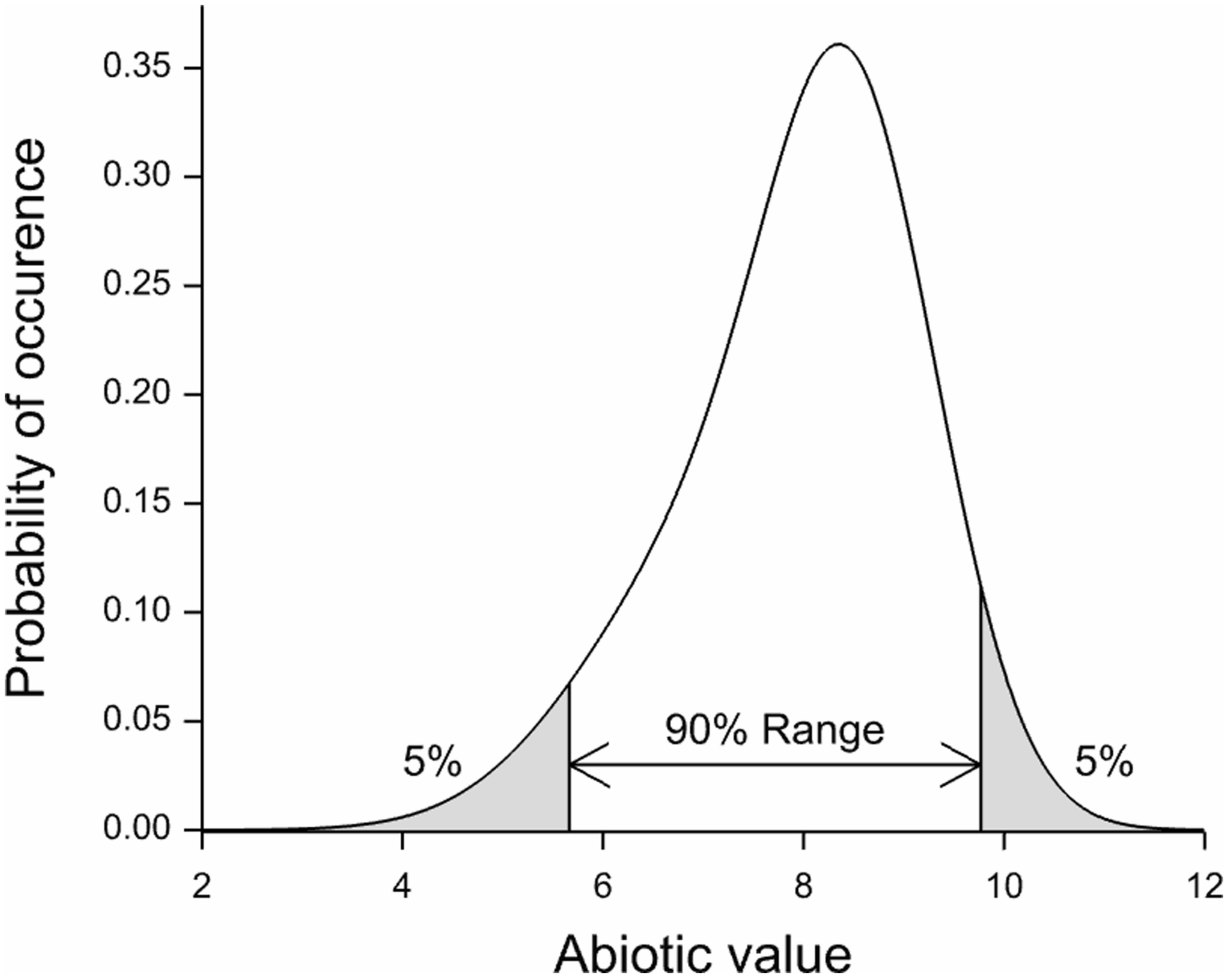
Hypothetical response curve (p-spline) for a hypothetical abiotic parameter, defining the range used in this research.

Responses per species were modelled by means of logistic regression employing a penalized B-spline [23] to estimate the curve (details in Wamelink *et al.*) [24]. Applying the spline function has the advantage that the responses of the species are independent of the number of findings. By dividing the abiotic (x) axes in 25 parts and subsequently calculating the chance of occurrence per part, a bias due to the number of findings is prevented. For each species an indicator value was derived as the mean of the response curve. We could have used the spline to estimate the range of the species. However, this would give ranges for only a limited number of species and based on a relative small number of relevés (just over 7000 for soil pH and over 1000 for the other abiotic parameters). The species indicator values were therefore used to calculate the mean abiotic values for a calibration dataset with 160,000 relevés, representative for the Dutch vegetation [25]. However, this was never tested and relevés were made for all kind of purposes and are therefore not random divided over Dutch nature. From the resulting responses for the abiotic parameters per species the 5 and 95 percentile were retrieved from a re-estimated species response curve, employing the full calibration set of 160,000 relevés, giving the range at which each species occurs (Figure 1). By applying this calibration dataset we are able to increase the number of species for which we can estimate a response. The length of the range of the species was defined as the difference between the 5 and 95 percentile of the response curve. The full method and the database used are described in Wamelink *et al.* [19], [22], [24].

To counteract the effect of non-random selection of the plots we used the 5 and 95 percentiles of the ranges of the fitted species response curve (Figure 1), instead of simply the abiotic value of the relevés at which the species occurs. To understand why this works consider the case in which relatively more narrow abiotic values are present in the database, or for a part of the abiotic range the plots are overrepresented. Using only the abiotic values for which a species is present will inevitably result in percentiles which are too narrow; the overrepresented plots will dominate the range. Since the effect of this might be different for the 5 and 95 percentile, this will also affect the range. However, the fitted species response curve, employing presence and absence data, will in principle not be affected by the non-random selection of the abiotic values as long as there are data for the full range of the abiotic values. Consequently, the range as estimated from the fitted curve will not be heavily affected.

### Rare species selection

The species list with responses was split in two: one part containing rare species and one containing the other species. The latter group will subsequently be called common species. Rare species were defined as species fulfilling the red list criteria [26] and as such indicated in the Dutch flora [27]. The rarity of the species is based on their frequency and trend as measured on the Dutch national 5 by 5 km grid, based on the period 1980–1990. There are five levels for the Dutch red list:

1. Red list category 0: species extinct in the Netherlands
2. Red list category 1; Species occurs in 1–12 grids, with a decrease in grid frequency of at least 50%, or species occurs in 13–40 grids, with a decrease in grid frequency of at least 75%.
3. Red list category 2: Species occurs in 1–12 grids, with a decrease in grid frequency of 25–50%, or species occurs in 13–40 grids, with a decrease in grid frequency of 50–75%, or the species occurs in 41–225 grids, with a decrease in frequency of at least 75%.
4. Red list category 3: the species occurs in 13–40 grids, with a decrease of 25–50%, or the species occurs in 41–225 grids, with a decrease of 25–75%.
5. Red list category 4: the species occurs in 1–60 grids, its occurrence is more or less stable and the species is not under immediate threat of becoming extinct.

All the species that fulfil one of the five above given criteria were merged into the set of rare species, in total 190 species out of 973 species.

### Statistical analysis

Means and standard errors for the ranges were calculated for the rare species group and the common species groups. A two sided student t-test was used to test for a difference between the averages of both groups for each abiotic soil parameter. All calculation, including the estimation of the spline functions were done in GenStat version 15 [28].

To test the results we looked within the red list species in order to establish whether ranges of species depend on the rarity of the species. To this end the red list species were divided into four pools: species that are not rare but made the list because of their decline (a), species that are rare (r), species that are very rare (rr) and species that are almost extinct (rrr, see Appendix S1, rarity is also based on Meijden [27]). Note: these categories were used in preference to the list above because the first list is a combination of rarity and the trend of a species, while the list presented here only is based on the rarity of the species and the effect of the latter is investigated here. To test for differences between the pools we simply ranked the average ranges of the four ‘rarity groups’ from one till four per abiotic parameter and then calculated the average ranking over the 23 abiotic parameters.

## Results

### Species responses

**Figure 2:**
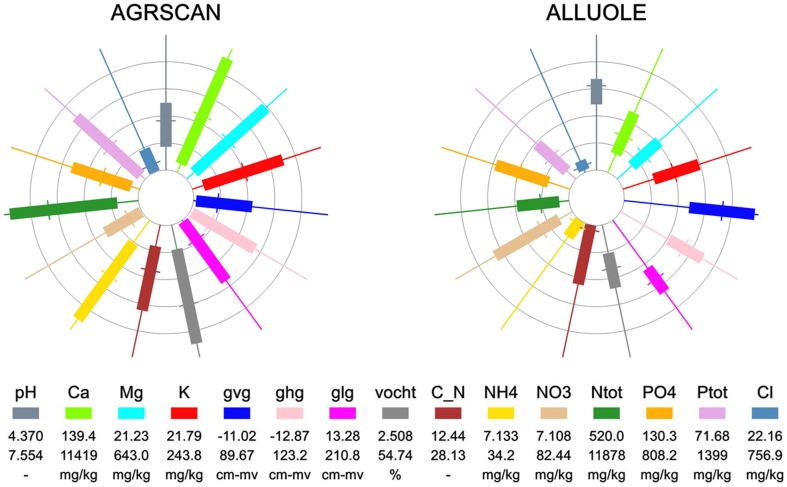
Abiotic ranges for *Agrostis canina* (brown bent, left) and *Allium oleraceum* (field garlic, right). The bar gives the range based on the 5 and 95 percentile of the occurrence of the species. Each spoke of the wheel represents a different abiotic parameter. The values per parameter are standardised with the minimum absolute value set to 0% and the maximum value set at 100%.The circles indicate the 0, 20th, 40th, 60th and 80th percentile. The wheels make it possible to compare the ranges between the species. With: pH: pH in water extract, Ca: calcium in water extract, Mg: magnesium in water extract, K: potassium in water extract, gvg: spring groundwater level, ghg: highest groundwater level, glg: lowest groundwater level, vocht: moist content of the soil, C_N: C/N ratio, NH4: ammonium content in CaCl2 extract, NO3: nitrate content in CaCl2 extract, Ntot: total nitrogen content, PO4: phosphate content, Ptot: total phosphor content and Cl: chloride content.

Species were only included in further analyses if ranges could be estimated for a minimum of 20 abiotic parameters. Responses were thus estimated for 973 species (783 common and 190 rare species, for an example see Figure 2). The newly designed figures give clearly the difference in ranges between the common species *Agrostis canina* (brown bent) and the rare species *Allium oleraceum* (field garlic) (Figure 2a versus 2b). The bars of the spokes of the wheels of the species show that the ranges (bars) for most of the abiotic parameters for *A. oleraceum* are narrower than for *A. canina*. 190 species fulfilled the red list criteria and were identified as rare species, leaving 783 common species for comparison.

### Differences in ranges

For nineteen out of the twenty-three abiotic soil parameters that were tested, the ranges for rare species were significantly narrower than for common species. No significant difference was found for the average highest and lowest groundwater table and for magnesium and organic matter content of the soil. Most of the differences in average range lay between 10 and 25% of the range (calculated against the average range of the rare species), including the important soil parameters pH, chloride ammonium concentration, potassium concentration and mean spring groundwater table. The biggest difference in range length is for nitrate concentration; up to 44%. The length of the range of a species may thus be an indicator for rare species.

Within the red list species, in general the rarer a species is, the narrower its range is likely to be (Appendix S2). The difference between the declining species ‘a’ and the rare species ‘r’ is narrow and not significant, but ranges for the common declining species ‘a’ is slightly narrower. All other possible combinations show statistically significant differences (Appendix S2). The difference between rare species in the category ‘rr’ and ‘rrr’ is bigger than the difference between ‘a’ and ‘r’. The species in the categories ‘rr’ and ‘rrr’ have, on average, clearly narrower ranges than both other categories. The rarest species (‘rrr’) have on average the narrowest ranges of all. In general, the rarer the species the narrower its range.

## Discussion

We clearly show that, on average, rare species have narrower ranges than common species for the majority of the examined abiotic soil parameters. None of the rare species showed significantly larger ranges. Obviously, this difference has a major impact on the species fundamental and realized niche and thus it occurrence. A wider habitat preference gives a species an in principle higher tolerance and therefore as well a higher resilience compared to species with narrower habitat preferences. This will be reflected in its spatial distribution, but also have an effect on the occurrence of the species as a result of natural changes of abiotic circumstances in time. The latter both within a season but also between years, since for both abiotic circumstances may change. Unfavourable circumstances during a part of the season or in some years may cause that species with narrow habitat requirement breadth cannot occur, whereas species with a wider habitat requirement breath can. For the most part, these ranges do not depend on the number of findings per species. The results therefore indicate that rare species have narrower habitat preferences for abiotic soil parameters than common species. Spitale [29] also found that rare spring bryophytes species had narrow habitat requirement breadth. Macandza *et al.* [30] found that for three wild grazer species of the African savannah, the rare sable antelope and the common buffalo and zebra, habitat requirement breadth and resource availability played a role in the difference in occurrence and consequently rarity of species. They concluded that rare species may persist by specializing and precisely selecting favourite patches to graze. Though these are animals, the results are similar to our results for plant species.

This research has been carried out for single responses; the interaction between abiotic parameters is not included, also due to lack of data. It is often speculated that interaction exists on a major scale [31]. Including interactions in relation to species responses may give different results; e.g. for a single response a species may have a wide range for soil pH but, for example, when phosphorus content is low it may only be able to grow at intermediate pH levels. How this will influence the results presented here is so far unknown to us. The biggest difference between rare and common species is present for nitrate concentration of the soil (44%). One of the major pressures on the occurrence of plant species in the Netherlands, but also in many other places in Europe, North America and Asia is an excessive nitrogen deposition [32], [33]. Nitrogen deposition leads to higher nitrogen concentrations in the soil and has all kinds of effects on plants [34]; some species benefit more than others by e.g. outgrowing them. We believe that our results indicate at least one of the reasons, why many rare species are threatened in the Netherlands: they have narrow ranges for nitrogen concentration and cannot cope with the input of anthropogenic nitrogen. Nitrate concentration in the soil is a result of release from biological processes (e.g. mineralisation and nitrification) and uptake by plants and bacteria and denitrification. This makes the nitrate concentration highly variable and thus resulting in a relative high uncertainty in the relation between plant species and their preferred nitrate concentration in the soil. However, this applies for both groups examined here. Furthermore we try to minimize this uncertainty by collecting as much data as possible, covering the whole growth season and under different environmental conditions (e.g. temperature, moist content of the soil, vegetation structure).

The occurrence of plant species depends on many factors, of which dispersal [11], [12], habitat availability [13], [14], [15], [16], grazing and vegetation management [16], [17] and abiotic conditions (including climate)[12], [16], [17], [18], [19], [20] are among the most important. It is known that some rare species besides narrow niches (as is shown here) also have poor dispersal capacity [11], [12]. However, how big the importance of the above mentioned factors is on the species occurrence remains unknown. This can probably only be established after a multivariate analyses applied on a database not only containing abiotic measurements but also information on the dispersal capacity of the species, the reaction of the species towards management and grazing. It may be clear however that the results presented here give a strong signal that abiotic habitat widths of rare species are of importance explaining their occurrence.

The results obtained here are relevant to the recent past and circumstances in relation to present climate. Changes in climate may give changes in species preferences and ranges, also due to interactions in relation to abiotic soil parameters, which are not included in the analyses. In principle it is possible that some rare species may benefit from climate change and thus become less rare; other species with broader ranges may become rare. However, in general we postulate that species with narrow abiotic preferences are likely to suffer more from changes in environmental circumstances than species with broad habitat requirements, because new (favourable) circumstances may be out of the current range of the rare species. In such situations resilience of species will be less and they will therefore have more difficulty in reacting to temporary or permanent changes.

The results have major implications in relation to management and nature development. They indicate that, in order to preserve rare species, management has to be focussed on the maintenance and creation of habitats fulfilling the specific requirements of rare species, both in space and time. An example of this may be the way excessive nitrogen has been mitigated in heathlands in the Netherlands. In the past sod cutting took place on a large scale, removing narrow scale differences in (for example) altitude, vegetation, light and moisture. This led to low (poor) nutrient levels, but also removed narrow scale variation from the fields, where common heather (*Calluna vulgaris*) recovered, but many other species did not. It is particularly important for ecosystem functioning [35] and ecosystem resilience [36] to conserve (abiotic) gradients within ecosystems. It will be easier to reach tipping points for rare species than for common species, making them more vulnerable to changes and as a consequence to local extinction.

## Supporting Information

Appendix S1 **Ranges per species for 23 abiotic soil parameters including rarity of the species, with c: common species but on the red list due to the trend, r: rare species and on the red list, rr: very rare species and on the red list and rrr: almost extinct and on the red list.**
(XLSX)

Appendix S2 **Average range of the red list species per rarity category and the ranking (1–4) of the category per abiotic factor including the overall averages of the abiotic parameters for the ranking.**
(XLSX)

## References

[1] Díaz S, Fargione J, Chapin FS, Tilman D (2006) Biodiversity Loss Threatens Human Well-Being. PLoS Biol 4: 1300–1305. 10.1371/journal.pbio.0040277 PMC1543691 16895442

[2] Tilman D, Reich PB, Knops JMH (2006) Biodiversity and ecosystem stability in a decade-long grassland experiment. Nature 441: 629–632. 16738658 10.1038/nature04742

[3] Butchart SHM, Walpole M, Collen B, Strien A van, Scharlemann JPW, et al (2010) Global Biodiversity: Indicators of recent declines. Science 328: 1164. 20430971 10.1126/science.1187512

[4] Fagerli H, Gauss M, Benedictow A, Griesfeller J, Jonson JE, et al.. (2010) Transboundary acidification, eutrophication and ground level ozone in Europe in 2009. EMEP Report 1/2011. Oslo: Norwegian Meteorological Institute.

[5] Reich PB, Knops J, Tilman D, Craine J, Ellsworth D, et al (2001) Plant diversity enhances ecosystem responses to elevated CO2 and nitrogen deposition. Nature 410: 809–812. 11298447 10.1038/35071062

[6] Bevill RL, Louda SM (1999) Comparisons of Related Rare and Common Species in the Study of Plant Rarity. Conservation Biology 13: 493–498.

[7] Prendergast JR, Quinn RM, Lawton JH, Eversham BC, Gibbons DW, et al (1993) Rare species, the coincidence of diversity hotspots and conservation strategies. Nature 365: 335–337.

[8] Tittensor DP, Mora C, Jetz W, Lotze HK, Ricard D, et al (2010) Global patterns and predictors of marine biodiversity across taxa. Nature 466: 1098–1101. 20668450 10.1038/nature09329

[9] Gravel D, Bell T, Barbera C, Bouvier T, Pommier T, et al (2011) Experimental niche evolution alters the strength of the diversity-productivity relationship. Nature 469: 89–92. 21131946 10.1038/nature09592

[10] Bakker JP, Berendse F (1999) Constraints in the restoration of ecological diversity in grassland and heathland communities. Trends in Ecology and Evolution 14: 63–68. 10234254 10.1016/s0169-5347(98)01544-4

[11] Ozinga WA, Schaminée JHJ, Bekker RM, Bonn S, Poschlod P, et al (2005) Predictability of plant species composition from environmental conditions is constrained by dispersal limitation. Oikos 108: 555–561.

[12] Normand S, Ricklefs RE, Skov F, Bladt J, Tackenberg O, et al (2011) Postglacial migration supplements climate in determining plant species ranges in Europe. Proceedings of the Royal Society B: Biological Sciences 278: 3644–3653. 21543356 10.1098/rspb.2010.2769 PMC3203492

[13] Foley JA, DeFries R, Asner GP, Barford C, Bonan G, et al (2005) Global Consequences of Land Use. Science 309: 570–574. 16040698 10.1126/science.1111772

[14] Potts SG, Biesmeijer JC, Kremen C, Neumann P, Schweiger O, et al (2010) Global pollinator declines: Trends, impacts and drivers. Trends in Ecology and Evolution 25: 345–353. 20188434 10.1016/j.tree.2010.01.007

[15] Condé S, Jones-Walters L, Torre-Marín A, Romao C (2010) EU 2010 Biodiversity Baseline. Copenhagen: EEA Technical report No 12/2010. EEA.

[16] Wesche K, Krause B, Culmsee H, Leuschner C (2012) Fifty years of change in Central European grassland vegetation: Large losses in species richness and animal-pollinated plants. Biological Conservation 150: 76–85.

[17] Ruprecht E, Enyedi MZ, Eckstein RL, Donath TW (2010) Restorative removal of plant litter and vegetation 40 years after abandonment enhances re-emergence of steppe grassland vegetation. Biological Conservation 143: 449–456.

[18] Austin MP, Niel KP van (2011) Improving species distribution models for climate change studies: Variable selection and scale. Journal of Biogeography 38: 1–8.

[19] Wamelink GWW, Goedhart PW, Dobben HF van, Berendse F (2005) Plant species as predictors of soil pH: replacing expert judgement by measurements. Journal of vegetation science 16: 461–470.

[20] Richardson PJ, MacDougall AS, Larson DW (2012) Fine-scale spatial heterogeneity and incoming seed diversity additively determine plant establishment. Journal of Ecology 100: 939–949.

[21] Braun-Blanquet J (1964) Pflanzensoziologie: Grundzüge der Vegetationskunde. 3. ed. Springer, Wien..

[22] Wamelink GWW, Adrichem MHC van, Dobben HF van, Frissel JY, Held M den, et al (2012) Vegetation relevés and soil measurements in the Netherlands; a database. Biodiversity and Ecology 2012: 125–132.

[23] Eilers HC, Marx BD (1996) Flexible smoothing with Bsplines and penalties. Statistical Science 11: 89–121.

[24] Wamelink GWW, Goedhart PW, Malinowska AH, Frissel JY, Wegman RJM, et al (2011) Ecological ranges for the pH and NO3 of syntaxa: A new basis for the estimation of critical loads for acid and nitrogen deposition. Journal of Vegetation Science 22: 741–749.

[25] Schaminee JHJ, Hennekens SM, Ozinga W (2012) The Dutch national vegetation database. Biodiversity and Ecology 2012: 201–209.

[26] Mace GM, Stuart SN (1994) Draft IUCN Red List Categories, version 2.2. Species 21–22: 13–24.

[27] Meijden R Van der (1996) Heukels flora van Nederland. Groningen: Wolters-Noordhoff.

[28] GenStat. Available: www.vsni.co.uk.

[29] Spitale D (2012) A comparative study of common and rare species in spring habitats. Ecoscience 19: 80–88.

[30] Macandza VA, Owen-Smith N, Cain JW (2012) Habitat and resource partitioning between abundant and relatively rare grazing ungulates. Journal of Zoology 287: 175–185.

[31] Laughlin DC, Abella SR (2007) Abiotic and biotic factors explain independent gradients of plant community composition in ponderosa pine forests. Ecological Modelling 205: 231–240.

[32] Galloway JN, Aber JD, Erisman JW, Seitzinger SP, Howarth RW, et al (2003) The Nitrogen Cascade. Bioscience 53: 341–356.

[33] Wamelink GWW, Knegt B de, Pouwels R, Schuiling C, Wegman RMA, et al (2013) Considerable environmental bottlenecks for the Habitats and Birds directives Species in the Netherlands. Biological Conservation 165: 43–53.

[34] Stevens CJ, Manning P, Berg LJL van den, Graaf MCC de, Wamelink GWW, et al (2011) Ecosystem responses to reduced and oxidised nitrogen inputs in European terrestrial habitats. Environmental Pollution 159: 665–676. 21215502 10.1016/j.envpol.2010.12.008

[35] Isbell F, Calcagno V, Hector A, Connolly J, Harpole WS, et al (2011) High plant diversity is needed to maintain ecosystem services. Nature 477: 199–201. 21832994 10.1038/nature10282

[36] Scheffer M, Bascompte J, Brock WA, Brovkin V, Carpenter SR, et al (2009) Early-warning signals for critical transitions. Nature 461: 53–59. 19727193 10.1038/nature08227

