# Correction: Why Some Plant Species Are Rare

**DOI:** 10.1371/journal.pone.0111293

**Published:** 2014-10-14

**Authors:** The PLOS ONE Staff

The first author’s name is incorrect. The correct name is: G. W. Wieger Wamelink.

The third author’s name is incorrect. The correct name is: Joep Y. Frissel.

The correct citation is: Wamelink GWW, Goedhart PW, Frissel JY (2014) Why Some Plant Species Are Rare. PLoS ONE 9(7): e102674. doi:10.1371/journal.pone.0102674
